# Hybrid Molecules Containing a 7-Chloro-4-aminoquinoline Nucleus and a Substituted 2-Pyrazoline with Antiproliferative and Antifungal Activity

**DOI:** 10.3390/molecules21080969

**Published:** 2016-07-27

**Authors:** Alba Montoya, Jairo Quiroga, Rodrigo Abonia, Marcos Derita, Maximiliano Sortino, Alfredo Ornelas, Susana Zacchino, Braulio Insuasty

**Affiliations:** 1 Grupo de Investigación de Compuestos Heterocíclicos, Departamento de Química, Universidad del Valle, A. A. 25360 Cali, Colombia; montoyaarias340@gmail.com (A.M.); jaiquir@gmail.com (J.Q.); rodrigo.abonia@correounivalle.edu.co (R.A.); 2 Área Farmacognosia, Facultad de Ciencias Bioquímicas y Farmacéuticas, Universidad Nacional de Rosario, Suipacha 531, 2000 Rosario, Argentina; mgderita@hotmail.com (M.D.); msortino@fbioyf.unr.edu.ar (M.S.); 3 Department of Chemistry, University of Texas at El Paso, 799689, 500 W. University Ave. El Paso, TX 79902, USA; aornelas3@miners.utep.edu

**Keywords:** cyclocondensation reaction, chalcones, *N-*aryl-2-pyrazolines, antifungal, antiproliferative activity

## Abstract

Twenty-four new hybrid analogues (**15**–**38**) containing 7-chloro-4-aminoquinoline and 2-pyrazoline N-heterocyclic fragments were synthesized. Twelve of the new compounds were evaluated against 58 human cancer cell lines by the U.S. National Cancer Institute (NCI). Compounds **25**, **30**, **31**, **36**, and **37** showed significant cytostatic activity, with the most outstanding GI_50_ values ranging from 0.05 to 0.95 µM. The hybrid compounds (**15**–**38**) were also evaluated for antifungal activity against *Candida albicans* and *Cryptococcus neoformans*. From the obtained results some structure–activity relationships were outlined.

## 1. Introduction

**Scheme 1:**
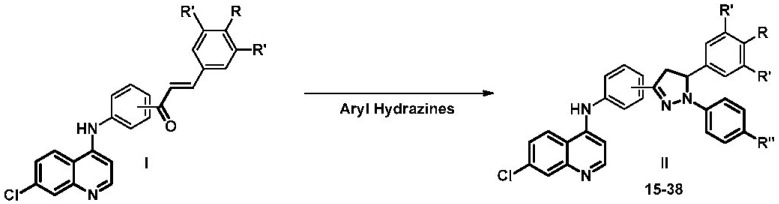
Designed synthetic strategy for the preparation of the target compounds **15**–**38**. Within structures, **I** represents the starting material with the 7-chloro-4-aminoquinoline nucleus and **II** is the desired product with the 2-pyrazoline moiety.

The substituted 2-pyrazoline moiety (**II**, Scheme 1) represents a structural component of significant interest in the field of Medicinal Chemistry, due to their prominent pharmacological effects, such as antimicrobial, antimycobacterial, antifungal, antiamoebic, anti-inflammatory, analgesic, antidepressant, and anticancer activities [1,2,3]. Compounds with this moiety also possess other biological properties like Nitric Oxide Synthase (NOS) inhibition and Cannabinoid CB1 receptor antagonism, among others [4].

On the other hand, structure–activity relationship studies on 4-aminoquinolines showed that the 7-chloro-4-aminoquinoline nucleus (**I**, Scheme 1) that is present in pharmacologically active substances displays a broad range of biological activities [5,6,7,8]. Therefore, the incorporation of this active pharmacophore into the structure of new heterocyclic compounds might improve their biological activity. 

Based on the above considerations and as a part of our current project devoted to the synthesis of novel nitrogen-containing heterocyclic compounds with biological activity [9,10,11,12,13], we decided to attempt the synthesis of hybrid compounds composed of both the 7-chloro-4-aminoquinoline nucleus and the substituted 2-pyrazoline moiety in a single structure (**15**–**38**, Scheme 1) looking for compounds with significant biological activities.

The synthesized compound were tested against an ample panel of cancer cell lines within the Developmental Therapeutics Program (DTP) at the National Cancer Institute (NCI) due to the fact that the moiety 2-pyrazoline [11,12] has demonstrated antitumor activities. Furthermore, considering that several antineoplastic compounds have proven valuable antifungal activities [14,15], we tested them as antifungals against two clinically important fungi, *Candida albicans* and *Cryptococcus neoformans*. It is important to take into account the limited number of efficacious antifungal drugs, which are not completely effective for the eradication of mycoses. There is, therefore, an urgent need for new antifungal chemical structures as alternatives to the existing ones [16].

## 2. Results and Discussion

### 2.1. Chemistry

**Scheme 2:**
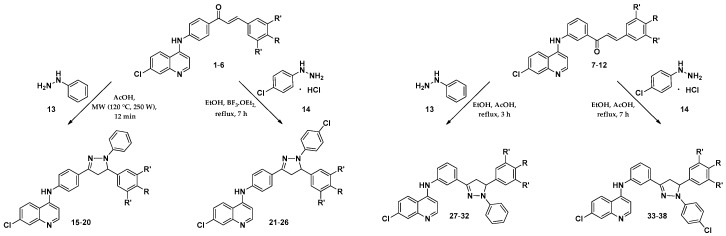
General scheme for the synthesis of new *N*-phenyl and *N*-4-chlorophenylpyrazolines **15**–**38** (methodology previously described in full in [15]).

The α,β-unsaturated carbonyl compounds **1**–**12** were prepared using a previously reported methodology (Scheme 2) [15]. These compounds were used as starting materials for the synthesis of the target products **15**–**38** (Scheme 2) of varied structures (**15**–**38**) that were obtained in good yields, as shown in Table 1.

Even though the precursors **1**–**12** for the synthesis of these pyrazolines are very similar, different reaction conditions had to be used for the synthesis of each particular series, as shown in Scheme 2. It was observed that the electron density of the substituents on the aryl ring next to the α,β-unsaturated ketone highly influenced the rates of the cyclocondensation reactions. Electron-donating groups (EDG) increased the reaction time, while electron-withdrawing groups (EWG) favored the reaction; this may be due to the fact that EWG increased the electrophilic character of the carbonyl carbon atom, while the EDG decreased it.

**Figure 1:**
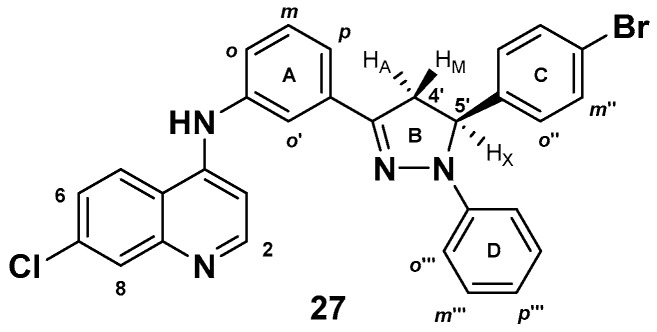
Structure of compound **27** showing the numbering of most carbons.

The effective cyclocondensation of the 1,3-dielectrophilic system (C=C-C=O) in compounds **1**–**12** was confirmed by means of NMR spectroscopy (see Experimental section). For the discussion, we will take *N*-phenyl pyrazoline **27** as an example (Figure 1).

In the ^1^H-NMR spectrum (DMSO-*d*_6_) of the compound **27**, the signals corresponding to the three chemically and magnetically non-equivalent protons of the pyrazoline ring **B** appeared as a spin coupling system AMX. Three double-doublets are observed at 3.13, 3.93, and 5.52 ppm. They belong to methylenic protons on the diastereotopic center C-4′ (H_A_ and H_M_) and to the methine proton H-5′ (H_X_) of the pyrazoline ring, with coupling constants of ^2^*J*_AM_ = 17.4, ^3^*J*_AX_ = 6.1 and ^3^*J*_MX_ = 12.1 Hz. Downfield, 13 signals with multiplicity according to their substitution pattern were assigned; eight of them correspond to the aromatic protons of the three aryl rings **A**, **C**, and **D** and the H-3 quinoline proton between 6.73 and 7.76 ppm; four signals between 7.59 and 8.49 ppm belong to the quinoline protons H-2, H-5, H-6, and H-8, and the N-H proton was observed at 9.19 ppm. Additionally, the 26 magnetically different carbons were found at their respective chemical shifts in the ^13^C-NMR spectrum of compound **27**.

In addition, the structures of compounds **15**–**38** were also confirmed through electron impact mass spectrometry (EIMS). The molecular ion peaks (M^+·^) of all compounds were observed at their respective molecular mass and the fragmentation pattern was in good agreement with the already reported for 2-pyrazoline derivatives [17]. Furthermore, the molecular ion peak of each compound was found to be the most stable fragment or base peak in 75% of the synthesized compounds. Mass spectrometry data on all the synthesized pyrazoline derivatives **15**–**38** are provided in the Experimental Section.

### 2.2. Antiproliferative Activity

As a preliminary screening, compounds **15**–**38** were submitted to the Developmental Therapeutics Program (DTP) at the National Cancer Institute (NCI) for evaluation of their antiproliferative activity against different human tumor cell lines. Twelve (**16**, **18**, **19**, **22**, **24**, **25**, **28**, **30**, **31**, **34**, **36**, and **37**) of the submitted compounds were selected and subjected to a preliminary evaluation against 58 tumor cell lines at a single dose of 10 µM and 48 h of incubation. The output from the single dose screening was reported as a mean graph available for analysis by the COMPARE program (data not shown). The results of this first assay showed that only compounds **25**, **30**, **31**, **36**, and **37** were active. It was observed that none of the pyrazolines containing EWG or weakly activating groups like methyl at R and R′ position (see Scheme 2) were active in the cancer cell lines.

The active compounds **25**, **30**, **31**, **36**, and **37** passed to a second evaluation step in order to determine their cytostatic activity against 58 tumor cell lines of leukemia, melanoma, lung, colon, brain, breast, ovary, kidney, and prostate. The results were expressed in the following parameters according to previously published protocols [18,19,20,21]: GI_50_, which is the molar concentration of the compounds required to inhibit 50% of the growth of cell lines (relative to untreated cells), and LC_50_, which is a parameter of cytotoxicity that reflects the molar concentration needed to kill 50% of the cells [22]. The active compounds were evaluated at five concentration levels (100, 10, 1.0, 0.1, and 0.01 µM) and the test consisted of a 48-h continuous drug exposure protocol using sulforhodamine B (SRB) protein assay to estimate cell growth [18,19,20,21]. As an interesting result, compounds **25**, **30**, **31**, **36**, and **37** exhibited significant cytostatic activity, with GI_50_ values lower than 1.0 × 10^−6^ M against several human cancer cell lines. The biological response elicited by these compounds could be attributed, in part, to the EDG on ring C (4-OCH_3_ and 3,4,5-triOCH_3_). With the exception of compound **31**, compounds **30**, **36**, and **37** were more active (in terms of the average number of cell lines) than compound **25**, which is presumably due to the *meta* substitution on ring **A**.

A comparison between the values of GI_50_ of compounds **25**, **30**, **31**, **36**, **37**, and the standard drug (adriamycin) against several tumor cell lines showed that all of these compounds have similar or even better cytostatic activity than the reference drug, as observed in Table 2. In comparison to adriamycin (GI_50_ = 0.12 μM), compound **30** showed better activity against the UACC-62 cell line with a GI_50_ = 0.05 μM. Compound **30** was also potent against KAKI-1 (GI_50_ = 0.68 μM) as compared to adriamycin (GI_50_ = 0.95 μM). Interestingly, all of the compounds previously mentioned (**25**, **30**, **31**, **36**, **37**) showed better cytostatic activity than adriamycin (GI_50_ = 6.46 μM) by a remarkable difference in GI_50_ values, going from 0.34 to 2.30 μM against HCT-15 tumor cell line. In Table 2, the compounds that exhibited promising cytostatic activity against different cell lines are highlighted in gray.

### 2.3. Antifungal Activity

In order to have a look into the potential usefulness of these compounds as candidates for the developments of new antifungal drugs, we investigated the antifungal properties of compounds **15**–**38** against two clinically important fungal species, *C. neoformans* and *C. albicans*. The selection of *C. neoformans* was due to the fact that this opportunistic fungus is the main cause of cryptococcal meningoencephalitis, which has a high incidence among HIV patients with impaired defenses [23]. High rates of fungal persistence and frequent disease relapse have motivated the search for new compounds that display antifungal properties against this fungus [24].

Moreover, *C.* *albicans* is the fourth leading cause of nosocomial bloodstream infection (BSI) in intensive care units, causing fatal invasive candidiasis in a high percentage of patients [25]. For this reason, the development of new potential anticandidal drugs is crucial. 

To assess antifungal activities, the standardized microbroth dilution method M-27A3 for yeasts of the Clinical and Laboratory Standards Institute was used [26]. Percentages of growth inhibition of each fungus were found using all compounds **15**–**38** with a concentration range within 250–3.9 µg/mL, which allowed for the determination of MIC_100_, MIC_80_, and MIC_50_.

For a more comprehensive analysis of the results, we grouped all compounds (**15**–**38**) into two series (i, ii) that differ only in the position of the diaryl–azole substituent on the **A** ring (Table 3): series (i) includes compounds **15**–**26** with the diaryl–azole moiety in the *p*-position of ring **A**; series (ii) includes compounds **27**–**38** with the same moiety in the *m*-position. Each series was sub-divided into two sub-groups, (i.1) and (ii.1), which comprise compounds with an un-substituted ring **D** (**15**–**20** and **27**–**32**, respectively), and (i.2) and (ii.2), which include compounds with a *p*-Cl substituted ring **D** (**21**–**26** and **33**–**38**, respectively). Table 3 shows the MIC_100_, MIC_80_, and MIC_50_ of each compound. It can be observed that MIC_50_ values displayed by several compounds (i.e., **25**, **32**, **36**) were highly promising.

**Figure 2:**
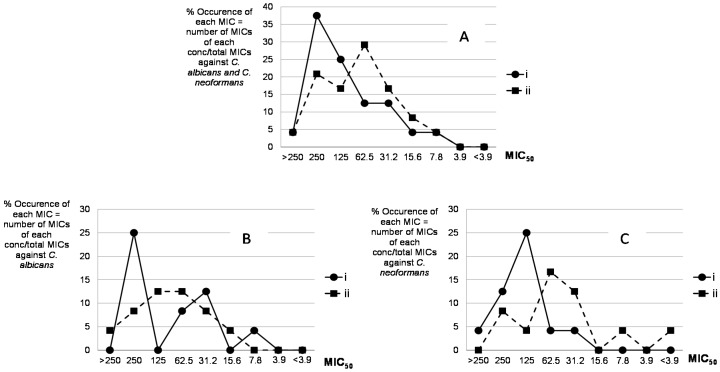
% Occurrence of each MIC value = number of MICs of each conc/total MICs acting against [(**A**) *C. albicans* + *C. neoformans* (*Ca + Cn*) (24 MIC_50_ values were considered 100%); (**B**) *C. albicans* (12 MIC_50_ values were considered 100%); (**C**) *C. neoformans* (12 MIC_50_ values were considered 100%]; (i) includes compounds **15**–**26** with the diaryl-azole moiety in the *p*-position of ring **A**; (ii) includes compounds **27**–**38** with the same moiety in the *m*-position.

To gain insight into the structure–activity relationships of the whole series, we compared first the antifungal behavior of all compounds of series (i) with those of series (ii) against both fungi. We focused on the last column (MIC_50_) of Table 3. This column contains 24 MIC_50_ values (against both fungi) for compounds **15**–**26** of group (i), and 24 MIC_50_ values of compounds **27**–**38** of group (ii). Then, the concentration values of MIC_50_ within group (i) (>250, 250, 125, 62.5, 31.2, 15.6. 7.8, 3.9, and <3.9 µg/mL) were analyzed to determine the number of times they were repeated within this series against both fungi and a percentage of occurrence of each MIC_50_ value was calculated (respective to the 24 total MIC_50_ values obtained in this group). The same analysis was applied to the compounds in group (ii). With these data, a comparative graph was produced (Figure 2A). Lastly, the antifungal properties of all compounds in series (i) (**15**–**26**) and those in series (ii) (**27**–**38**) were analyzed against each fungus separately; these data are presented in Figure 2B,C.

As can be seen in Figure 2A, within group (i) 40% of all MIC_50_ values fall into the value 250 µg/mL, while no compounds produced MIC_50_ values of 7.8 µg/mL or lower. Instead, 21% of the compounds within group (ii) fall into the 250 µg/mL concentration, while 4% fall into 7.8 and <3.9 µg/mL. From these results, it is clear that compounds of series (ii) exhibit better antifungal properties than those of series (i) against both fungi. Regarding the behavior against each fungus separately, series (ii) showed a higher percentage of lower MIC_50_ values over (i) against *C. neoformans* (Figure 2C). Also, the same trend can be observed against *C. albicans*, where compounds of series (i) showed a much higher percentage of MIC_50_ values at 250 µg/mL than compounds in series (ii). These results indicate that the position of the azole moiety does play a role in the antifungal activity and that compounds with this moiety in the *m*-position are better antifungal candidates than those with the azole moiety in the *p*-position.

**Figure 3:**
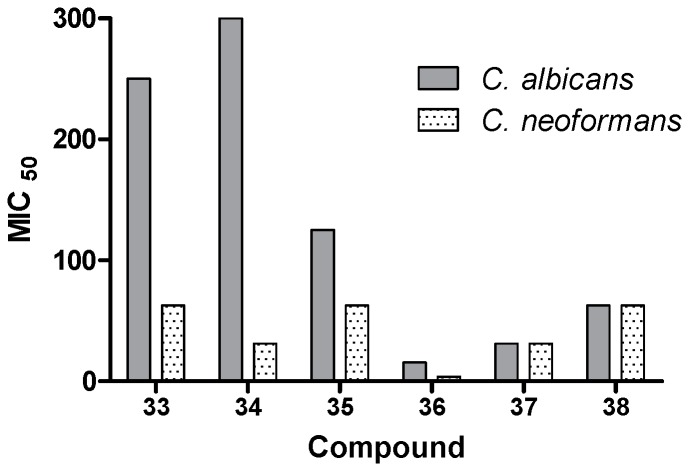
Comparison of MIC_50_ values of each compound of the (ii.2) sub-series against *C. albicans* or *C. neoformans.*

From the previous analyses, it was determined that within the whole series of hybrids of *N*-aryl-substituted pyrazolines with 7-chloro-4-aminoquinoline nucleus tested (**15**–**38**), compounds of the sub-series (ii.2) with the aryl-azole moiety in *m*-position of ring **A** and a *p*-Cl substituted ring **D** were the most active, mainly against *C. neoformans*. To corroborate the higher antifungal activity of these (ii-2) compounds against *C. neoformans* over *C. albicans*, a MIC_50_ values’ comparison of **33**–**38** against both fungi is shown in Figure 3.

As can be observed in Figure 3, the MIC_50_ values of compounds **33**–**38** in *C. neoformans* are much lower than those exhibited by C. *albicans*, thus suggesting a higher sensitivity of *C. neoformans* against this group of compounds. In this figure, it is evidenced that among the compounds of (ii.2) sub-group, compound **36** was by far the most active one, mainly against *C. neoformans*, and thus it is the best anti-cryptococcal candidate of the compounds in this work.

## 3. Materials and Methods

### 3.1. General Information

Commercially available starting materials, reagents, and solvents were used as supplied. Microwave irradiation reactions were performed in glass vessels (10 mL) using a CEM Discover Focused Microwave Synthesis System™ apparatus (Matthews, NC, USA), with power output from 0 to 300 W. TLC analyses were performed on Merck (EMD Millipore, Billerica, MA, USA) silica gel 60 F254 aluminum plates. Melting points were determined in a Büchi (Instrumart, South Burlington, VT, USA) melting point apparatus and are uncorrected. IR spectra were performed on a Shimadzu (Scientific Instruments, Seattle, WA, USA) FTIR 8400 spectrophotometer in KBr disks. The ^1^H- and ^13^C-NMR spectra were run on a Bruker (Karlsruhe, Germany) DPX 400 spectrophotometer operating at 400 MHz and 100 MHz respectively, using dimethylsulfoxide-*d*_6_ as solvents and tetramethylsilane as internal reference. The mass spectra were obtained on a Hewlett Packard (Scientific Instrument Services, Ringoes, NJ, USA) HP Engine-5989 spectrometer (equipped with a direct inlet probe) operating at 70 eV. The elemental analyses were obtained using a Thermo-Finnigan Flash EA1112 CHN (Elemental Microanalysis Ltd., (Okehampton, Devon, UK) elemental analyzer.

### 3.2. Synthesis

#### 3.2.1. General Procedure for the Synthesis of the Precursors **1**–**12**

Using the same previously reported method [15], precursors **1**–**12** were obtained. 

#### 3.2.2. General Procedure for the Preparation of Compounds **15**–**20**

A mixture of 4-(7-chloroquinolin-4-yl) amino chalcone 1–6 (0.11 mmol), phenylhydrazine **13** (0.33 mmol) in glacial acetic acid (10 mL) was submitted to microwave irradiation for 12 min at 250 W and 120 °C. Once the reaction mixture was cooled to room temperature, the resulting solution was neutralized with concentrated ammonium hydroxide. Then, crushed ice was added to the solution and a solid was precipitated, collected by vacuum filtration, washed thoroughly with water, dried, and recrystallized from ethanol.

*N-(4-(5-(4-Bromophenyl)-1-phenyl-4,5-dihydro-1H-pyrazol-3-yl)phenyl)-7-chloroquinolin-4-amine* (**15**). Yellow solid; 90% yield; mp: 97–99 °C. FTIR (KBr) υ(cm^−1^): 3350 (NH), 3054 (=C-H), 1599 and 1576 (C=N and C=C). ^1^H-NMR (400 MHz, DMSO-*d*_6_) δ ppm 3.12 (dd, *J* = 17.4, 6.1 Hz, 1H, H-4′a), 3.92 (dd, *J* = 17.4, 12.1 Hz, 1H, H-4′b), 5.49 (dd, *J* = 12.1, 6.1 Hz, 1H, H-5′), 6.72 (t, *J* = 7.6 Hz, 1H, Ar-H), 6.99 (d, *J* = 7.6 Hz, 2H, Ar-H), 7.07 (d, *J* = 5.4 Hz, 1H, H-3), 7.12–7.19 (m, 2H, Ar-H), 7.26 (d, *J* = 8.4 Hz, 2H, Ar-H), 7.41 (d, *J* = 8.7 Hz, 2H, Ar-H), 7.54 (d, *J* = 8.4 Hz, 2H, Ar-H), 7.59 (dd, *J* = 9.1, 2.3 Hz, 1H, H-6), 7.77 (d, *J* = 8.7 Hz, 2H, Ar-H), 7.92 (d, *J* = 2.3 Hz, 1H, H-8), 8.42 (d, *J* = 9.1 Hz, 1H, H-5), 8.51 (d, *J* = 5.4 Hz, 1H, H-2), 9.24 (br s, 1H, NH). ^13^C-NMR (100 MHz, DMSO-*d*_6_) δ ppm 43.3 (CH_2_), 63.0, 100.0 (C), 103.3, 113.4, 119.1 (C), 119.2, 120.9 (C), 122.2, 125.0, 125.7, 127.4, 127.9 (C), 128.0, 128.7, 129.4, 132.4, 131.9 (C), 134.6 (C), 142.4 (C), 144.6 (C), 147.6 (C), 149.5 (C), 152.0. MS (70 eV) *m*/*z* (%): 552 (100, M^+^), 397 (17), 368 (34), 313 (32), 271 (35), 236 (47), 123 (34), 98 (47), 91 (67), 83 (54), 57 (81), 44 (67). Anal. Calcd. For C_30_H_22_BrClN_4_: C, 65.05; H, 4.00; N, 10.12. Found: C, 65.20; H, 3.98; N, 10.15.

*7-Chloro-N-(4-(5-(4-chlorophenyl)-1-phenyl-4,5-dihydro-1H-pyrazol-3-yl)phenyl)quinolin-4-amine* (**16**). Yellow solid; 92% yield; mp: 121–124 °C. FTIR (KBr) υ(cm^−1^): 3263 (NH), 3056 (=C-H), 1597 and 1572 (C=N and C=C). ^1^H-NMR (400 MHz, DMSO-*d*_6_) δ ppm 3.09 (dd, *J* = 17.4, 6.1 Hz, 1H, H-4′a), 3.90 (dd, *J* = 17.4, 11.9 Hz, 1H, H-4′b), 5.48 (dd, *J* = 11.9, 6.1 Hz, 1H, H-5′), 6.71 (t, *J* = 7.5 Hz, 1H, Ar-H), 6.98 (d, *J* = 7.5 Hz, 2H, Ar-H), 7.06 (d, *J* = 5.1 Hz, 1H, H-3), 7.15 (t, *J* = 7.5 Hz, 2H, Ar-H), 7.31 (d, *J* = 8.5 Hz, 2H, Ar-H), 7.36–7.44 (m, 4H, Ar-H), 7.57 (dd, *J* = 9.0, 2.1 Hz, 1H, H-6), 7.76 (d, *J* = 8.5 Hz, 2H, Ar-H), 7.91 (d, *J* = 2.1 Hz, 1H, H-8), 8.41 (d, *J* = 9.0 Hz, 1H, H-5), 8.50 (d, *J* = 5.1 Hz, 1H, H-2), 9.25 (br s, 1H, NH). ^13^C-NMR (100 MHz, DMSO-*d*_6_) δ ppm 42.9 (CH_2_), 62.4, 63.1 (C), 102.8, 112.9, 118.6, 121.6, 124.5, 125.2, 126.9, 127.3 (C), 127.6, 127.9, 128.9, 129.0, 131.9 (C), 134.1 (C), 140.9 (C), 141.5 (C), 144.2 (C), 147.1 (C), 147.2 (C), 149.5 (C), 152.0. MS (70 eV) *m*/*z* (%): 508 (100, M^+^), 397 (19), 368 (9), 279 (23), 254 (15), 243 (14), 91 (47), 77 (17). Anal. Calcd. For C_30_H_22_Cl_2_N_4_: C, 70.73; H, 4.35; N, 11.00. Found: C, 70.79; H, 4.37; N, 10.89.

*7-Chloro-N-(4-(1,5-diphenyl-4,5-dihydro-1H-pyrazol-3-yl)phenyl)quinolin-4-amine* (**17**). Yellow solid; 83% yield; mp: 109–111 °C. FTIR (KBr) υ(cm^−1^): 3270 (NH), 3060 (=C-H), 1598 and 1573 (C=N and C=C). ^1^H-NMR (400 MHz, DMSO-*d*_6_) δ ppm 3.11 (dd, *J* = 17.4, 6.2 Hz, 1H, H-4′a), 3.93 (dd, *J* = 17.4, 12.2 Hz, 1H, H-4′b), 5.48 (dd, *J* = 12.2, 6.2 Hz, 1H, H-5′), 6.65–6.73 (m, 2H, Ar-H), 7.00 (d, *J* = 7.8 Hz, 2H, Ar-H), 7.06–7.18 (m, 3H, Ar-H and H-3), 7.23–7.38 (m, 4H, Ar-H) 7.42 (d, *J* = 8.6 Hz, 2H, Ar-H), 7.60 (dd, *J* = 9.1, 2.2 Hz, 1H, H-6), 7.78 (d, *J* = 8.6 Hz, 2H, Ar-H), 7.92 (d, *J* = 2.2 Hz, 1H, H-8), 8.43 (d, *J* = 9.1 Hz, 1H, H-5), 8.52 (d, *J* = 5.3 Hz, 1H, H-2), 9.27 (s, 1H, NH). ^13^C-NMR (100 MHz, DMSO-*d*_6_) δ ppm 43.6 (CH_2_), 63.6, 112.5, 113.3, 118.8, 122.1, 125.1, 125.6, 126.4, 127.4, 127.9, 128.2, 129.2, 129.4, 134.5 (C), 141.4 (C), 143.1 (C), 144.8 (C), 147.5 (C), 147.8 (C), 150.0 (C), 150.1 (C), 152.5, 169.5 (C). MS (70 eV) *m*/*z* (%): 474 (100, M^+^), 397 (19), 279 (15), 121 (35), 105 (68), 91 (42), 77 (52), 57 (33), 43 (31). Anal. Calcd. For C_30_H_23_ClN_4_: C, 75.86; H, 4.88; N, 11.80. Found: C, 75.81; H, 4.65; N, 11.73.

*7-Chloro-N-(4-(5-(4-methoxyphenyl)-1-phenyl-4,5-dihydro-1H-pyrazol-3-yl)phenyl)quinolin-4-amine* (**18**) [27]. Yellow solid; 77% yield; mp: 111–112 °C. FTIR (KBr) υ(cm^−1^): 3312 (NH), 3040 (=C-H), 1597 and 1572 (C=N and C=C). ^1^H-NMR (400 MHz, DMSO-*d*_6_) δ ppm 3.06 (dd, *J* = 17.3, 6.2 Hz, 1H, H-4′a), 3.70 (s, 3H, OCH_3_), 3.87 (dd, *J* = 17.3, 12.0 Hz, 1H, H-4′b), 5.40 (dd, *J* = 12.0, 6.2 Hz, 1H, H-5′), 6.69 (t, *J* = 7.3 Hz, 1H, Ar-H), 6.88 (d, *J* = 8.8 Hz, 2H, Ar-H), 7.00 (d, *J* = 8.0 Hz, 2H, Ar-H), 7.06 (d, *J* = 5.3 Hz, 1H, H-3), 7.10–7.17 (m, 2H, Ar-H), 7.20 (d, *J* = 8.5 Hz, 2H, Ar-H), 7.40 (d, *J* = 8.5 Hz, 2H, Ar-H), 7.57 (dd, *J* = 9.0, 1.9 Hz, 1H, H-6), 7.76 (d, *J* = 8.5 Hz, 2H, Ar-H), 7.91 (d, *J* = 1.9 Hz, 1H, H-8), 8.40 (d, *J* = 9.0 Hz, 1H, H-5), 8.49 (d, *J* = 5.3 Hz, 1H, H-2), 9.25 (br, 1H, NH). ^13^C-NMR (100 MHz, DMSO-*d*_6_) δ ppm 43.5 (CH_2_), 55.5 (OCH_3_), 63.1, 103.1, 113.4, 114.5, 114.8, 118.9 (C), 119.0, 122.3, 124.8, 125.8, 127.3, 127.6, 128.2 (C), 129.4, 134.8 (C), 134.9 (C), 141.0 (C), 144.7 (C), 147.4 (C), 148.1 (C), 149.5 (C), 152.3, 158.9 (C). MS (70 eV) *m*/*z* (%): 504 (100, M^+^), 399 (10), 279 (18), 121 (18), 91 (34), 77 (13), 57 (10). Anal. Calcd. For C_31_H_25_ClN_4_O: C, 73.73; H, 4.99; N, 11.09. Found: C, 73.42; H, 4.87; N, 11.07.

*7-Chloro-N-(4-(1-phenyl-5-(3,4,5-trimethoxyphenyl)-4,5-dihydro-1H-pyrazol-3-yl)phenyl)quinolin-4-amine* (**19**). Yellow solid; 76% yield; mp: 99–102 °C. FTIR (KBr) υ(cm^−1^): 3240 (NH), 3056 (=C-H), 1598 and 1575 (C=N and C=C). ^1^H-NMR (400 MHz, DMSO-*d*_6_) δ ppm 3.15 (dd, *J* = 17.4, 7.3 Hz, 1H, H-4′a), 3.63 (s, 3H, OCH_3_), 3.71 (s, 6H, 2 × OCH_3_), 3.91 (dd, *J* = 17.4, 12.1 Hz, 1H, H-4′b), 5.34 (dd, *J* = 12.1, 7.3 Hz, 1H, H-5′), 6.64 (s, 2H, Ar-H), 6.74 (t, *J* = 7.2 Hz, 1H, Ar-H), 7.02–7.10 (m, 3H, Ar-H and H-3), 7.15–7.21 (m, 2H, Ar-H), 7.42 (d, *J* = 8.8 Hz, 2H, Ar-H), 7.60 (dd, *J* = 9.1, 2.2 Hz, 1H, H-6), 7.79 (d, *J* = 8.8 Hz, 2H, Ar-H), 7.92 (d, *J* = 2.2 Hz, 1H, H-8), 8.43 (d, *J* = 9.1 Hz, 1H, H-5), 8.52 (d, *J* = 5.3 Hz, 1H, H-2), 9.25 (s, 1H, NH). ^13^C-NMR (100 MHz, DMSO-*d*_6_) δ ppm 43.7 (CH_2_), 56.4 (2 × OCH_3_), 60.5 (OCH_3_), 64.4, 103.5, 113.6, 119.2, 120.2 (C), 122.1, 125.1, 125.7, 127.5, 127.9 (C), 128.2 (C), 128.8 (C), 129.4, 132.2, 133.3 (C), 134.6 (C), 137.1, 139.0 (C), 141.4 (C), 145.4 (C), 147.9 (C), 152.4, 153.8 (C). MS (70 eV) *m*/*z* (%): 564 (100, M^+^), 397 (35), 279 (18), 243 (12), 91 (32), 77 (12). Anal. Calcd. For C_33_H_29_ClN_4_O_3_: C, 70.14; H, 5.17; N, 9.92. Found: C, 70.09; H, 5.11; N, 9.82.

*7-Chloro-N-(4-(1-phenyl-5-(p-tolyl)-4,5-dihydro-1H-pyrazol-3-yl)phenyl)quinolin-4-amine* (**20**). Yellow solid; 86% yield; mp: 89–92 °C. FTIR (KBr) υ(cm^−1^): 3230 (NH), 3052 (=C-H), 1599 and 1577 (C=N and C=C). ^1^H-NMR (400 MHz, DMSO-*d*_6_) δ ppm 2.25 (s, 3H, CH_3_), 3.07 (dd, *J* = 17.3, 6.3 Hz, 1H, H-4′a), 3.89 (dd, *J* = 17.3, 12.2 Hz, 1H, H-4′b), 5.41 (dd, *J* = 12.2, 6.3 Hz, 1H, H-5′), 6.66–6.72 (m, 1H, Ar-H), 7.00 (d, *J* = 7.8 Hz, 2H, Ar-H), 7.07 (d, *J* = 5.3 Hz, 1H, H-3), 7.11–7.15 (m, 4H, Ar-H), 7.17–7.21 (m, 2H, Ar-H), 7.41 (d, *J* = 8.6 Hz, 2H, Ar-H), 7.58 (dd, *J* = 9.0, 2.2 Hz, 1H, H-6), 7.77 (d, *J* = 8.6 Hz, 2H, Ar-H), 7.92 (d, *J* = 2.2 Hz, 1H, H-8), 8.43 (d, *J* = 9.0 Hz, 1H, H-5), 8.51 (d, *J* = 5.3 Hz, 1H, H-2), 9.24 (s, 1H, NH). ^13^C-NMR (100 MHz, DMSO-*d*_6_) δ ppm 21.2 (CH_3_), 43.6 (CH_2_), 63.5, 103.3, 112.6, 113.4, 118.9, 122.1, 125.3, 126.3, 127.3, 128.0 (C), 128.2, 129.2, 130.0, 134.5 (C), 137.0 (C), 140.1 (C), 141.4 (C), 144.5 (C), 147.5 (C), 147.7 (C), 149.9 (C), 150.2 (C), 152.5. MS (70 eV) *m*/*z* (%): 488 (11, M^+^), 368 (31), 236 (47), 210 (61), 150 (60), 108 (100), 97 (49), 83 (60), 69 (56), 43 (39). Anal. Calcd. For C_31_H_25_ClN_4_: C, 76.14; H, 5.15; N, 11.46. Found: C, 76.12; H, 5.09; N, 11.36.

#### 3.2.3. General Procedure for the Preparation of Compounds **21**–**26**

A mixture of the corresponding chalcone 1–6 (0.11 mmol), 4-chlorophenylhydrazine hydrochloride 14 (0.22 mmol), BF_3_·OEt_2_ (0.2 mL, molar excess) as catalyst, and EtOH (8 mL) was heated under reflux for 7 h. After this time, the product was observed as a precipitate and was filtered and washed three times with the EtOH/H_2_O (1:0.5) mixture. No further purification was required. 

*N-(4-(5-(4-Bromophenyl)-1-(4-chlorophenyl)-4,5-dihydro-1H-pyrazol-3-yl)phenyl)-7-chloroquinolin-4-amine* (**21**). Yellow solid; 80% yield; mp: >300 °C. FTIR (KBr) υ(cm^−1^): 3410 (NH), 3020 (=C-H), 1613 and 1589 (C=N and C=C). ^1^H-NMR (400 MHz, DMSO-*d*_6_) δ ppm 3.18 (dd, *J* = 17.5, 5.8 Hz, 1H, H-4′a), 3.97 (dd, *J* = 17.5, 12.1 Hz, 1H, H-4′b), 5.59 (dd, *J* = 12.1, 5.8 Hz, 1H, H-5′), 6.94 (d, *J* = 6.7 Hz, 1H, H-3), 7.00 (d, *J* = 8.9 Hz, 2H, Ar-H), 7.21 (d, *J* = 8.9 Hz, 2H, Ar-H), 7.30 (d, *J* = 8.3 Hz, 2H, Ar-H), 7.42 (d, *J* = 8.3 Hz, 2H, Ar-H), 7.54 (d, *J* = 8.4 Hz, 2H, Ar-H), 7.84 (dd, *J* = 9.2, 1.3 Hz, 1H, H-6), 7.89 (d, *J* = 8.4 Hz, 2H, Ar-H), 8.16 (d, *J* = 1.3 Hz, 1H, H-8), 8.55 (d, *J* = 6.7 Hz, 1H, H-2), 8.86 (d, *J* = 9.2 Hz, 1H, H-5), 11.07 (br s, 1H, NH). ^13^C-NMR (100 MHz, DMSO-*d*_6_) δ ppm 43.3 (CH_2_), 62.9, 101.5, 114.9, 117.0 (C), 120.9, 123.0 (C), 125.2, 126.5, 127.6, 127.7, 128.4, 129.3, 129.6, 130.8 (C), 132.6 (C), 138.3 (C), 138.5 (C), 141.1 (C), 141.4 (C), 143.2 (C), 145.2, 148.0 (C), 154.0 (C). MS (70 eV) *m*/*z* (%): 586 (55, M^+^), 368 (24), 346 (28), 313 (33), 236 (31), 125 (37), 97 (53), 71 (59), 57 (100), 43 (94). Anal. Calcd. For C_30_H_21_BrCl_2_N_4_: C, 61.25; H, 3.60; N, 9.52. Found: C, 61.15; H, 3.78; N, 9.63. 

*N-(4-(1,5-bis(4-Chlorophenyl)-4,5-dihydro-1H-pyrazol-3-yl)phenyl)-7-chloroquinolin-4-amine* (**22**). Yellow solid; 87% yield; mp: >300 °C. FTIR (KBr) υ(cm^−1^): 3418 (NH), 3025 (=C-H), 1614 and 1569 (C=N and C=C). ^1^H-NMR (400 MHz, DMSO-*d*_6_) δ ppm 3.19 (dd, *J* = 17.6, 5.7 Hz, 1H, H-4′a), 3.97 (dd, *J* = 17.6, 12.2 Hz, 1H, H-4′b), 5.59 (dd, *J* = 12.2, 5.7 Hz, 1H, H-5′), 6.95 (d, *J* = 6.8 Hz, 1H, H-3), 7.01 (d, *J* = 8.8 Hz, 2H, Ar-H), 7.21–7.26 (m, 4H, Ar-H), 7.53–7.57 (m, 4H, Ar-H), 7.83–7.96 (m, 3H, Ar-H and H-6), 8.11 (d, *J* = 1.4 Hz, 1H, H-8), 8.56 (d, *J* = 6.8 Hz, 1H, H-2), 8.80 (d, *J* = 9.2 Hz, 1H, H-5), 11.00 (br s, 1H, NH). ^13^C-NMR (100 MHz, DMSO-*d*_6_) δ ppm 43.3 (CH_2_), 63.0, 101.3, 114.9, 116.8 (C), 120.4, 121.2 (C), 123.0 (C), 125.5, 126.4, 127.8, 127.9, 128.7, 129.3, 131.1 (C), 132.5, 138.1 (C), 138.8 (C), 140.2 (C), 141.7 (C), 143.1 (C), 144.7, 147.9 (C), 154.6 (C). MS (70 eV) *m*/*z* (%): 542 (100, M^+^), 431 (15), 368 (13), 279 (20), 125 (14). Anal. Calcd. For C_30_H_21_Cl_3_N_4_: C, 66.25; H, 3.89; N, 10.30. Found: C, 66.40; H, 4.00; N, 10.52. 

*7-Chloro-N-(4-(1-(4-chlorophenyl)-5-phenyl-4,5-dihydro-1H-pyrazol-3-yl)phenyl)quinolin-4-amine* (**23**). Yellow solid; 73% yield; mp: >300 °C. FTIR (KBr) υ(cm^−1^): 3429 (NH), 3021 (=C-H), 1613 and 1591 (C=N and C=C). ^1^H-NMR (400 MHz, DMSO-*d*_6_) δ ppm 3.18 (dd, *J* = 17.5, 6.0 Hz, 1H, H-4′a), 3.98 (dd, *J* = 17.5, 12.3 Hz, 1H, H-4′b), 5.56 (dd, *J* = 12.3, 6.0 Hz, 1H, H-5′), 6.94 (d, *J* = 6.8 Hz, 1H, H-3), 7.01 (d, *J* = 8.9 Hz, 2H, Ar-H), 7.20 (d, *J* = 8.9 Hz, 2H, Ar-H), 7.24–7.32 (m, 3H, Ar-H), 7.35 (d, *J* = 7.2 Hz, 2H, Ar-H), 7.55 (d, *J* = 8.4 Hz, 2H, Ar-H), 7.85 (dd, *J* = 9.0, 1.9 Hz, 1H, H-6), 7.91 (d, *J* = 8.4 Hz, 2H, Ar-H), 8.16 (d, *J* = 1.9 Hz, 1H, H-8), 8.55 (d, *J* = 6.8 Hz, 1H, H-2), 8.90 (d, *J* = 9.0 Hz, 1H, H-5), 11.17 (br s, 1H, NH). ^13^C-NMR (100 MHz, DMSO-*d*_6_) δ ppm 43.6 (CH_2_), 63.7, 101.6, 114.9, 117.1 (C), 121.3, 122.8 (C), 125.0, 126.3, 126.5, 127.5, 127.6, 128.1, 129.2, 129.6, 130.7 (C), 136.4 (C), 138.1 (C), 138.6 (C), 142.5 (C), 143.4 (C), 145.5, 147.9 (C), 153.6 (C). MS (70 eV) *m*/*z* (%): 508 (100, M^+^), 431 (16), 369 (12), 279 (20), 254 (17), 125 (35), 90 (14). Anal. Calcd. For C_30_H_22_Cl_2_N_4_: C, 70.73; H, 4.35; N, 11.00. Found: C, 70.69; H, 4.42; N, 10.95. 

*7-Chloro-N-(4-(1-(4-chlorophenyl)-5-(4-methoxyphenyl)-4,5-dihydro-1H-pyrazol-3-yl)phenyl)quinolin-4-amine* (**24**). Yellow solid; 67% yield; mp: >300 °C. FTIR (KBr) υ(cm^−1^): 3421 (NH), 2679 (=C-H), 1616 and 1597 (C=N and C=C). ^1^H-NMR (400 MHz, DMSO-*d*_6_) δ ppm 3.15 (dd, *J* = 17.6, 5.8 Hz, 1H, H-4′a), 3.72 (s, 3H, OCH_3_), 3.94 (dd, *J* = 17.6, 12.1 Hz, 1H, H-4′b), 5.51 (dd, *J* = 12.1, 5.8 Hz, 1H, H-5′), 6.86–6.97 (m, 3H, Ar-H and H-3), 7.02 (d, *J* = 8.8 Hz, 2H, Ar-H), 7.18–7.21 (m, 4H, Ar-H), 7.54 (d, *J* = 8.4 Hz, 2H, Ar-H), 7.82–7.95 (m, 3H, Ar-H and H-6), 8.14 (d, *J* = 1.6 Hz, 1H, H-8), 8.55 (d, *J* = 6.8 Hz, 1H, H-2), 8.83 (d, *J* = 9.0 Hz, 1H, H-5), 11.06 (br s, 1H, NH). ^13^C-NMR (100 MHz, DMSO-*d*_6_) δ ppm 43.5 (CH_2_), 55.6 (OCH_3_), 63.2, 101.4, 114.9, 115.0, 116.9 (C), 120.6, 122.7 (C), 125.4, 126.4, 127.6, 127.7, 127.8, 129.2, 131.2 (C), 134.3 (C), 138.1 (C), 138.6 (C), 140.6 (C), 143.3 (C), 145.0, 147.8 (C), 154.3 (C), 159.1 (C). MS (70 eV) *m*/*z* (%): 538 (100, M^+^). Anal. Calcd. For C_31_H_24_Cl_2_N_4_O: C, 69.02; H, 4.48; N, 10.39. Found: C, 69.08; H, 4.44; N, 10.51. 

*7-Chloro-N-(4-(1-(4-chlorophenyl)-5-(3,4,5-trimethoxyphenyl)-4,5-dihydro-1H-pyrazol-3-yl)phenyl)quinolin-4-amine* (**25**). Yellow solid; 63% yield; mp: 218–220 °C. FTIR (KBr) υ(cm^−1^): 3434 (NH), 2969 (=C-H) and 1595 (C=N and C=C). ^1^H-NMR (400 MHz, DMSO-*d_6_*) δ ppm 3.22 (dd, *J* = 17.6, 6.9 Hz, 1H, H-4′a), 3.64 (s, 3H, OCH_3_), 3.72 (s, 6H, 2 × OCH_3_), 3.96 (dd, *J* = 17.6, 12.1 Hz, 1H, H-4′b), 5.42 (dd, *J* = 12.1, 6.9 Hz, 1H, H-5′), 6.95 (d, *J* = 7.0 Hz, 1H, H-3), 7.05 (d, *J* = 9.0 Hz, 2H, Ar-H), 7.21–7.29 (m, 4H, Ar-H), 7.55 (d, *J* = 8.6 Hz, 2H, Ar-H), 7.86 (dd, *J* = 9.0, 1.8 Hz, 1H, H-6), 7.91 (d, *J* = 8.6 Hz, 2H, Ar-H), 8.15 (d, *J* = 1.8 Hz, 1H, H-8), 8.55 (d, *J* = 7.0 Hz, 1H, H-2), 8.85 (d, *J* = 9.0 Hz, 1H, H-5), 11.08 (br s, 1H, NH). ^13^C-NMR (100 MHz, DMSO-*d*_6_) δ ppm 43.7 (CH_2_), 56.4 (2 × OCH_3_), 60.5 (OCH_3_), 64.4, 101.3, 103.4, 114.8, 115.1, 116.9 (C), 122.8 (C), 123.0 (C), 125.3, 126.5, 127.0, 127.7, 129.2, 131.1 (C), 137.2 (C), 138.2 (C), 140.2 (C), 141.0 (C), 143.8 (C), 144.7, 145.4 (C), 148.2 (C), 153.9 (C). MS (70 eV) *m*/*z* (%): 598 (7, M^+^), 420 (100), 294 (43), 281 (21), 166 (20), 125 (39). Anal. Calcd. For C_33_H_28_Cl_2_N_4_O_3_: C, 66.11; H, 4.71; N, 9.35. Found: C, 66.13; H, 4.94; N, 9.36. 

*7-Chloro-N-(4-(1-(4-chlorophenyl)-5-(p-tolyl)-4,5-dihydro-1H-pyrazol-3-yl)phenyl)quinolin-4-amine* (**26**). Yellow solid; 70% yield; mp: 296–298 °C. FTIR (KBr) υ(cm^−1^): 3424 (NH), 2974 (=C-H), 1614 and 1590 (C=N and C=C). ^1^H-NMR (400 MHz, DMSO-*d*_6_) δ ppm 2.26 (s, 3H, CH_3_), 3.15 (dd, *J* = 17.6, 5.9 Hz, 1H, H-4′a), 3.95 (dd, *J* = 17.6, 12.3 Hz, 1H, H-4′b), 5.51 (dd, *J* = 12.3, 5.9 Hz, 1H, H-5′), 6.94 (d, *J* = 6.8 Hz, 1H, H-3), 7.01 (d, *J* = 9.2 Hz, 2H, Ar-H), 7.12–7.24 (m, 6H, Ar-H), 7.55 (d, *J* = 8.6 Hz, 2H, Ar-H), 7.85 (dd, *J* = 9.1, 2.0 Hz, 1H, H-6), 7.90 (d, *J* = 8.6 Hz, 2H, Ar-H), 8.18 (d, *J* = 2.0 Hz, 1H, H-8), 8.55 (d, *J* = 6.8 Hz, 1H, H-2), 8.89 (d, *J* = 9.1 Hz, 1H, H-5), 11.05 (br s, 1H, NH). ^13^C-NMR (100 MHz, DMSO-*d*_6_) δ ppm 21.2 (CH_3_), 43.5 (CH_2_), 63.5, 101.3, 114.9, 116.9 (C), 120.3, 122.8 (C), 125.4, 126.3, 126.6, 127.6, 127.7, 129.2, 130.1, 131.2 (C), 137.3 (C), 138.1 (C), 138.6 (C), 139.5 (C), 140.4 (C), 143.3 (C), 144.5, 147.8 (C), 154.4 (C). MS (70 eV) *m*/*z* (%): 522 (100, M^+^), 279 (20), 243 (13), 125 (38). Anal. Calcd. For C_31_H_24_Cl_2_N_4_: C, 71.13; H, 4.62; N, 10.70. Found: C, 71.09; H, 4.56; N, 10.71.

#### 3.2.4. General Procedure for the Preparation of Compounds **27**–**32**

A mixture of chalcone 7–12 (0.11 mmol), phenylhydrazine **13** (0.22 mmol), glacial acetic acid (0.8 mL, molar excess) and EtOH (8 mL) was heated under reflux for 3 h until complete consumption of the chalcone (monitored by TLC). The product was observed as a precipitate and was filtered and washed with EtOH/H_2_O (1:0.5) mixture. No further purification was required. 

*N-(3-(5-(4-Bromophenyl)-1-phenyl-4,5-dihydro-1H-pyrazol-3-yl)phenyl)-7-chloroquinolin-4-amine* (**27**). Yellow solid; 83% yield; mp: 228–231 °C. FTIR (KBr) υ(cm^−1^): 3354 (NH), 3053 (=C-H), 1602 and 1569 (C=N and C=C). ^1^H-NMR (400 MHz, DMSO-*d*_6_) δ ppm 3.13 (dd, *J* = 17.4, 6.1 Hz, 1H, H-4′a), 3.93 (dd, *J* = 17.4, 12.1 Hz, 1H, H-4′b), 5.52 (dd, *J* = 12.1, 6.1 Hz, 1H, H-5′), 6.73 (t, *J* = 7.6 Hz, 1H, Ar-H), 6.98–7.03 (m, 3H, Ar-H and H-3), 7.16 (t, *J* = 7.6 Hz, 2H, Ar-H), 7.25 (d, *J* = 8.3 Hz, 2H, Ar-H), 7.39–7.41 (m, 1H, Ar-H), 7.47 (d, *J* = 4.5 Hz, 2H, Ar-H), 7.54 (d, *J* = 8.3 Hz, 2H, Ar-H), 7.59 (dd, *J* = 9.0, 1.7 Hz, 1H, H-6), 7.76 (s, 1H, Ar-H), 7.91 (d, *J* = 1.7 Hz, 1H, H-8), 8.44 (d, *J* = 9.0 Hz, 1H, H-5), 8.49 (d, *J* = 5.0 Hz, 1H, H-2), 9.19 (br s, 1H, NH). ^13^C-NMR (100 MHz, DMSO-*d*_6_) δ ppm 43.2 (CH_2_), 63.0, 79.7 (C), 102.6, 113.5, 119.4, 119.9, 121.0 (C), 122.0, 123.1, 124.9, 125.6, 128.2, 128.7, 129.5, 130.2, 132.4, 134.0 (C), 134.5 (C), 135.9 (C), 141.1 (C), 144.4 (C), 147.5 (C), 148.3 (C), 150.1 (C), 152.5. MS (70 eV) *m*/*z* (%): 552 (100, M^+^), 397 (62), 280 (40), 218 (24), 91 (33), 77 (45). Anal. Calcd. For C_30_H_22_BrClN_4_: C, 65.05; H, 4.00; N, 10.12. Found: C, 65.05; H, 4.11; N, 10.25. 

*7-Chloro-N-(3-(5-(4-chlorophenyl)-1-phenyl-4,5-dihydro-1H-pyrazol-3-yl)phenyl)quinolin-4-amine* (**28**). Yellow solid; 81% yield; mp: 187–188 °C. FTIR (KBr) υ(cm^−1^): 3263 (NH), 3056 (=C-H), 1597 and 1569 (C=N and C=C). ^1^H-NMR (400 MHz, DMSO-*d*_6_) δ ppm 3.13 (dd, *J* = 17.6, 6.0 Hz, 1H, H-4′a), 3.94 (dd, *J* = 17.6, 12.1 Hz, 1H, H-4′b), 5.55 (dd, *J* = 12.1, 6.0 Hz, 1H, H-5′), 6.73 (t, *J* = 7.2 Hz, 1H, Ar-H), 6.97–6.99 (m, 3H, Ar-H and H-3), 7.12–7.20 (m, 2H, Ar-H), 7.32 (d, *J* = 8.3 Hz, 2H, Ar-H), 7.37–7.43 (m, 3H, Ar-H), 7.48 (d, *J* = 4.8 Hz, 2H, Ar-H), 7.59 (dd, *J* = 8.8, 1.6 Hz, 1H, H-6), 7.77 (s, 1H, Ar-H), 7.91 (d, *J* = 1.6 Hz, 1H, H-8), 8.44 (d, *J* = 8.8 Hz, 1H, H-5), 8.50 (d, *J* = 5.3 Hz, 1H, H-2), 9.19 (br s, 1H, NH). ^13^C-NMR (100 MHz, DMSO-*d*_6_) δ ppm 42.7 (CH_2_), 62.5, 76.5 (C), 102.1, 113.0, 118.9, 119.4, 121.5 (C), 122.6, 124.5, 125.1, 127.7, 127.9, 129.0, 129.8, 132.8, 133.5 (C), 134.0 (C), 135.9 (C), 140.6 (C), 143.9 (C), 147.8 (C), 148.5, 148.9 (C), 149.6 (C), 152.1. MS (70 eV) *m*/*z* (%): 508 (100, M^+^), 397 (45), 296 (36), 281 (28), 218 (59), 43 (51). Anal. Calcd. For C_30_H_22_Cl_2_N_4_: C, 70.73; H, 4.35; N, 11.00. Found: C, 70.77; H, 4.39; N, 10.91. 

*7-Chloro-N-(3-(1,5-diphenyl-4,5-dihydro-1H-pyrazol-3-yl)phenyl)quinolin-4-amine* (**29**). Yellow solid; 73% yield; mp: 202–203 °C. FTIR (KBr) υ(cm^−1^): 3342 (NH), 3051 (=C-H), 1598 and 1567 (C=N and C=C). ^1^H-NMR (400 MHz, DMSO-*d*_6_) δ ppm 3.12 (dd, *J* = 17.5, 6.3 Hz, 1H, H-4′a), 3.94 (dd, *J* = 17.5, 12.2 Hz, 1H, H-4′b), 5.50 (dd, *J* = 12.2, 6.3 Hz, 1H, H-5′), 6.71 (t, *J* = 7.3 Hz, 1H, Ar-H), 6.97–7.03 (m, 3H, Ar-H and H-3), 7.10–7.17 (m, 2H, Ar-H), 7.23–7.41 (m, 6H, Ar-H), 7.45–7.49 (m, 2H, Ar-H), 7.59 (dd, *J* = 9.0, 2.0 Hz, 1H, H-6), 7.78 (s, 1H, Ar-H), 7.91 (d, *J* = 2.0 Hz, 1H, H-8), 8.45 (d, *J* = 9.0 Hz, 1H, H-5), 8.49 (d, *J* = 5.3 Hz, 1H, H-2), 9.19 (br s, 1H, NH). ^13^C-NMR (100 MHz, DMSO-*d*_6_) δ ppm 43.0 (CH_2_), 63.2, 99.5 (C), 102.1, 113.0, 118.4 (C), 119.4, 121.5, 122.6, 124.5, 125.1, 125.3, 125.8, 127.6, 128.9, 129.0, 129.1, 129.7, 133.6 (C), 134.0 (C), 142.4 (C), 144.1 (C), 146.8 (C), 147.9 (C), 149.5 (C), 152.0. MS (70 eV) *m*/*z* (%): 474 (100, M^+^), 397 (77), 280 (21), 218 (17), 91 (35), 77 (40). Anal. Calcd. For C_30_H_23_ClN_4_: C, 75.86; H, 4.88; N, 11.80. Found: C, 75.78; H, 4.92; N, 11.85. 

*7-Chloro-N-(3-(5-(4-methoxyphenyl)-1-phenyl-4,5-dihydro-1H-pyrazol-3-yl)phenyl)quinolin-4-amine* (**30**). Yellow solid; 68% yield; mp: 189–191 °C. FTIR (KBr) υ(cm^−1^): 3274 (NH), 3057 (=C-H), 1620 and 1599 (C=N and C=C). ^1^H-NMR (400 MHz, DMSO-*d*_6_): δ ppm = 3.09 (dd, *J* = 17.3, 6.2 Hz, 1H, H-4′a), 3.71 (s, 3H, OCH_3_), 3.90 (dd, *J* = 17.3, 12.2 Hz, 1H, H-4′b), 5.45 (dd, *J* = 12.2, 6.2 Hz, 1H, H-5′), 6.71 (t, *J* = 7.3 Hz, 1H, Ar-H), 6.89 (d, *J* = 8.7 Hz, 2H, Ar-H), 6.97–7.03 (m, 3H, Ar-H and H-3), 7.11–7.17 (m, 2H, Ar-H), 7.21 (d, *J* = 8.7 Hz, 2H, Ar-H), 7.39–7.41 (m, 1H, Ar-H), 7.45–7.48 (m, 2H, Ar-H), 7.57–7.60 (m, 1H, Ar-H), 7.77 (s, 1H, Ar-H), 7.91 (d, *J* = 2.0 Hz, 1H, H-8), 8.45 (d, *J* = 9.0 Hz, 1H, H-5), 8.49 (d, *J* = 5.3 Hz, 1H, H-2), 9.19 (s, 1H, NH). ^13^C-NMR (100 MHz, DMSO-*d*_6_): δ ppm = 43.0 (CH_2_), 55.0 (OCH_3_), 62.8, 102.1, 113.1, 114.3, 118.7, 119.3, 121.5, 122.5, 124.4, 125.0, 127.1, 127.7, 128.8, 129.7, 133.7 (C), 134.0 (C), 134.3 (C), 135.9 (C), 140.6 (C), 144.1 (C), 146.8 (C), 147.8 (C), 149.6 (C), 152.1, 158.5 (C). MS (70 eV) *m*/*z* (%): 504 (100, M^+^), 397 (57), 280 (25), 218 (21), 77 (29). Anal. Calcd. For C_31_H_25_ClN_4_O: C, 73.73; H, 4.99; N, 11.09. Found: C, 73.67; H, 4.89; N, 11.16.

*7-Chloro-N-(3-(1-phenyl-5-(3,4,5-trimethoxyphenyl)-4,5-dihydro-1H-pyrazol-3-yl)phenyl)quinolin-4-amine* (**31**). Yellow solid; 65% yield; mp: 183–186 °C. FTIR (KBr) υ(cm^−1^): 3285 (NH), 3028 (=C-H), 1665 (C=N and C=C). ^1^H-NMR (400 MHz, DMSO-*d*_6_) δ ppm 3.16 (dd, *J* = 17.5, 7.3 Hz, 1H, H-4′a), 3.72 (s, 3H, OCH_3_), 3.86 (s, 6H, 2 × OCH_3_), It is not observed (dd, 1H, H-4′b), 5.37 (dd, *J* = 12.2, 7.3 Hz, 1H, H-5′), 6.72–6.77 (m, 1H, Ar-H), 6.98–7.07 (m, 3H, Ar-H), 7.10–7.21 (m, 4H, Ar-H), 7.36–7.42 (m, 1H, Ar-H), 7.45–7.51 (m, 2H, Ar-H), 7.59 (dd, *J* = 9.1, 2.2 Hz, 1H, H-6), 7.78 (s, 1H, Ar-H), 7.92 (d, *J* = 2.2 Hz, 1H, H-8), 8.40–8.54 (m, 2H, H-5 and H-2), 9.62 (br s, 1H, NH). ^13^C-NMR (100 MHz, DMSO-*d*_6_) δ ppm 43.6 (CH_2_), 56.3 (2 x OCH_3_), 60.4 (OCH_3_), 64.4, 100.0 (C), 102.5, 113.7, 119.3 (C), 119.4, 119.8, 121.0 (C), 122.0, 123.1, 125.0, 125.6, 128.1, 129.4, 129.5, 130.3, 134.1 (C), 134.5 (C), 137.0 (C), 138.8 (C), 145.1 (C), 147.7 (C), 148.3 (C), 150.1 (C), 152.5. MS (70 eV) *m*/*z* (%): 564 (100, M^+^), 397 (65), 279 (14), 91 (37), 77 (14). Anal. Calcd. For C_33_H_29_ClN_4_O_3_: C, 70.14; H, 5.17; N, 9.92. Found: C, 69.97; H, 5.25; N, 10.03.

*7-Chloro-N-(3-(1-phenyl-5-(p-tolyl)-4,5-dihydro-1H-pyrazol-3-yl)phenyl)quinolin-4-amine* (**32**). Yellow solid; 72% yield; mp: 210–212 °C. FTIR (KBr) υ(cm^−1^): 3187 (NH), 3058 (=C-H), 1617 and 1594 (C=N and C=C). ^1^H-NMR (400 MHz, DMSO-*d*_6_) δ ppm 2.30 (s, 3H, CH_3_), 3.11 (dd, *J* = 17.4, 6.2 Hz, 1H, H-4′a), 3.91 (dd, *J* = 17.4, 12.2 Hz, 1H, H-4′b), 5.48 (dd, *J* = 12.2, 6.2 Hz, 1H, H-5′), 6.75 (t, *J* = 7.1 Hz, 1H, Ar-H), 6.83 (d, *J* = 7.0 Hz, 1H, H-3), 7.16–7.24 (m, 2H, Ar-H), 7.24–7.29 (m, 2H, Ar-H), 7.41 (d, *J* = 7.9 Hz, 2H, Ar-H), 7.57 (d, *J* = 7.9 Hz, 2H, Ar-H), 7.77–7.82 (m, 1H, Ar-H), 7.85 (d, *J* = 8.3 Hz, 2H, Ar-H), 7.87 (dd, *J* = 9.2, 2.2 Hz, 1H, H-6), 7.90 (s, 1H, Ar-H), 8.21 (d, *J* = 2.2 Hz, 1H, H-8), 8.52 (d, *J* = 7.0 Hz, 1H, H-2), 8.92 (d, *J* = 9.2 Hz, 1H, H-5), 9.19 (br s, 1H, NH). ^13^C-NMR (100 MHz, DMSO-*d*_6_) δ ppm 13.4 (CH_3_), 43.0 (CH_2_), 63.0, 100.9, 113.4, 114.5, 119.6, 119.7, 121.5, 122.4, 124.7, 125.0, 126.8, 127.8, 129.4, 130.4, 133.7 (C), 135.9 (C), 137.6 (C), 138.9 (C), 139.6 (C), 139.8 (C), 141.7 (C), 143.8, 144.1 (C), 146.3 (C), 155.5 (C). MS (70 eV) *m*/*z* (%): 488 (78, M^+^), 490 (100), 397 (52), 280 (23), 218 (19), 77 (14). Anal. Calcd. For C_31_H_25_ClN_4_: C, 76.14; H, 5.15; N, 11.46. Found: C, 76.10; H, 5.15; N, 11.52.

#### 3.2.5. General Procedure for the Preparation of Compounds **33**–**38**

A mixture of the corresponding 3-(7-chloroquinolin-4-yl)amino chalcone 7–12 (0.11 mmol), 4-chlorophenylhydrazine hydrochloride **14** (0.22 mmol), glacial acetic acid (0.2 mL, molar excess), and EtOH (8 mL) was heated under reflux for 8 h, until the reaction is complete and the product precipitated. Afterward, the solid product was filtered, washed with water, and recrystallized from ethanol.

*N-(3-(5-(4-Bromophenyl)-1-(4-chlorophenyl)-4,5-dihydro-1H-pyrazol-3-yl)phenyl)-7-chloroquinolin-4-amine* (**33**). Yellow solid; 75% yield; mp: 201–203 °C. FTIR (KBr) υ(cm^−1^): 3166 (NH), 3049 (=C-H), 1618 and 1595 (C=N and C=C). ^1^H-NMR (400 MHz, DMSO-*d*_6_) δ ppm 3.18 (dd, *J* = 17.6, 6.0 Hz, 1H, H-4′a), 3.96 (dd, *J* = 17.6, 12.4 Hz, 1H, H-4′b), 5.59 (dd, *J* = 12.4, 6.0 Hz, 1H, H-5′), 6.86 (d, *J* = 6.9 Hz, 1H, H-3), 6.99 (d, *J* = 8.8 Hz, 2H, Ar-H), 7.17–7.26 (m, 4H, Ar-H), 7.51 (d, *J* = 7.8 Hz, 1H, Ar-H), 7.55 (d, *J* = 8.3 Hz, 2H, Ar-H), 7.62 (t, *J* = 7.8 Hz, 1H, Ar-H), 7.74 (d, *J* = 7.8 Hz, 1H, Ar-H), 7.85–7.92 (m, 2H, Ar-H and H-6), 8.21 (d, *J* = 2.0 Hz, 1H, H-8), 8.53 (d, *J* = 6.9 Hz, 1H, H-2), 8.91 (d, *J* = 9.0 Hz, 1H, H-5), 11.28 (br s, 1H, NH). ^13^C-NMR (100 MHz, DMSO-*d*_6_) δ ppm 42.8 (CH_2_), 62.4, 100.5, 114.5, 116.0 (C), 119.3, 122.4, 122.6 (C), 124.9, 125.6, 126.2, 127.4, 127.8, 128.8, 129.1, 130.3, 132.1 (C), 133.8 (C), 137.5 (C), 138.4 (C), 139.2 (C), 140.7 (C), 142.5 (C), 143.5, 147.3 (C), 154.8 (C). MS (70 eV) *m*/*z* (%): 588 (100), 586 (84, M^+^), 431 (52), 279 (32), 243 (26), 218 (20), 125 (68), 111 (25), 90 (34). Anal. Calcd. For C_30_H_21_BrCl_2_N_4_: C, 61.25; H, 3.60; N, 9.52. Found: C, 61.32; H, 3.76; N, 10.01. 

*N-(3-(1,5-bis(4-Chlorophenyl)-4,5-dihydro-1H-pyrazol-3-yl)phenyl)-7-chloroquinolin-4-amine* (**34**). Yellow solid; 71% yield; mp: 206–207 °C. FTIR (KBr) υ(cm^−1^): 3160 (NH), 3049 (=C-H), 1617 and 1588 (C=N and C=C). ^1^H-NMR (400 MHz, DMSO-*d*_6_) δ ppm 3.17 (dd, *J* = 17.6, 6.0 Hz, 1H, H-4′a), 3.96 (dd, *J* = 17.6, 12.2 Hz, 1H, H-4′b), 5.60 (dd, *J* = 12.2, 6.0 Hz, 1H, H-5′), 6.85 (d, *J* = 7.0 Hz, 1H, H-3), 6.98 (d, *J* = 9.0 Hz, 2H, Ar-H), 7.19 (d, *J* = 9.0 Hz, 2H, Ar-H), 7.29 (d, *J* = 8.5 Hz, 2H, Ar-H), 7.41 (d, *J* = 8.5 Hz, 2H, Ar-H), 7.50 (dd, *J* = 8.0, 0.9 Hz, 1H, Ar-H), 7.61 (t, *J* = 8.0 Hz, 1H, Ar-H), 7.73 (d, *J* = 8.0 Hz, 1H, Ar-H), 7.84–7.91 (m, 2H, Ar-H and H-6), 8.21 (d, *J* = 2.0 Hz, 1H, H-8), 8.53 (d, *J* = 7.0 Hz, 1H, H-2), 8.93 (d, *J* = 9.0 Hz, 1H, H-5), 11.30 (br s, 1H, NH). ^13^C-NMR (100 MHz, DMSO-*d*_6_) δ ppm 41.2 (CH_2_), 49.8, 100.0 (C), 100.6, 116.5 (C), 119.8, 120.4 (C), 125.2, 126.6, 128.0, 128.1, 130.5, 130.7, 131.0, 131.2, 131.4, 132.4, 137.6 (C), 137.7 (C), 138.1 (C), 138.9 (C), 139.1 (C), 139.6 (C), 144.1, 155.4 (C), 198.2 (C). MS (70 eV) *m*/*z* (%): 542 (100, M^+^), 431 (49), 279 (26), 125 (18), 90 (13). Anal. Calcd. For C_30_H_21_Cl_3_N_4_: C, 66.25; H, 3.89; N, 10.30. Found: C, 66.27; H, 3.95; N, 10.36. 

*7-Chloro-N-(3-(1-(4-chlorophenyl)-5-phenyl-4,5-dihydro-1H-pyrazol-3-yl)phenyl)quinolin-4-amine* (**35**). Yellow solid; 61% yield; mp: 245–246 °C. FTIR (KBr) υ(cm^−1^): 3354 (NH), 3056 (=C-H), 1615 and 1591 (C=N and C=C). ^1^H-NMR (400 MHz, DMSO-*d*_6_) δ ppm 3.17 (dd, *J* = 17.6, 6.1 Hz, 1H, H-4′a), 3.97 (dd, *J* = 17.6, 12.3 Hz, 1H, H-4′b), 5.58 (dd, *J* = 12.3, 6.1 Hz, 1H, H-5′), 6.88 (d, *J* = 6.9 Hz, 1H, H-3), 7.00 (d, *J* = 9.0 Hz, 2H, Ar-H), 7.19 (d, *J* = 9.0 Hz, 2H, Ar-H), 7.28 (m, 3H, Ar-H), 7.33–7.39 (m, 2H, Ar-H), 7.50 (dd, *J* = 8.0, 0.8 Hz, 1H, Ar-H), 7.61 (t, *J* = 7.8 Hz, 1H, Ar-H), 7.72 (d, *J* = 7.8 Hz, 1H, Ar-H), 7.86 (dd, *J* = 9.1, 2.0 Hz, 1H, H-6), 7.90 (s, 1H, Ar-H), 8.18 (d, *J* = 2.0 Hz, 1H, H-8), 8.54 (d, *J* = 6.9 Hz, 1H, H-2), 8.89 (d, *J* = 9.1 Hz, 1H, H-5), 11.14 (br s, 1H, NH). ^13^C-NMR (100 MHz, DMSO-*d*_6_) δ ppm 43.5 (CH_2_), 63.6, 101.1, 114.9, 116.7 (C), 120.5, 122.6, 122.9 (C), 125.1, 125.8, 126.3, 126.6, 127.7, 128.1, 129.2, 129.6, 130.8, 134.3 (C), 138.3 (C), 138.5 (C), 140.5 (C), 142.3 (C), 143.2 (C), 144.8, 147.7 (C), 154.7 (C). MS (70 eV) *m*/*z* (%): 508 (100, M^+^), 431 (28), 279 (16), 236 (24), 125 (33), 111 (26), 97 (19), 83 (22), 57 (35), 43 (27). Anal. Calcd. For C_30_H_22_Cl_2_N_4_: C, 70.73; H, 4.35; N, 11.00. Found: C, 70.64; H, 4.38; N, 11.12. 

*7-Chloro-N-(3-(1-(4-chlorophenyl)-5-(4-methoxyphenyl)-4,5-dihydro-1H-pyrazol-3-yl)phenyl)quinolin-4-amine* (**36**). Yellow solid; 47% yield; mp: 216–218 °C. FTIR (KBr) υ(cm^−1^): 3189 (NH), 3060 (=C-H), 1617 and 1594 (C=N and C=C). ^1^H-NMR (400 MHz, DMSO-*d*_6_) δ ppm 3.20 (dd, *J* = 17.6, 7.0 Hz, 1H, H-4′a), 3.71 (s, 3H, OCH_3_), 3.95 (dd, *J* = 17.6, 12.3 Hz, 1H, H-4′b), 5.43 (dd, *J* = 12.3, 7.0 Hz, 1H, H-5′), 6.89 (d, *J* = 7.0 Hz, 1H, H-3), 6.98 (d, *J* = 9.0 Hz, 2H, Ar-H), 7.04 (d, *J* = 9.0 Hz, 2H, Ar-H), 7.22 (d, *J* = 8.8 Hz, 2H, Ar-H), 7.35 (d, *J* = 8.8 Hz, 2H, Ar-H), 7.51 (d, *J* = 8.0 Hz, 1H, Ar-H), 7.64 (t, *J* = 8.0 Hz, 1H, Ar-H), 7.76 (d, *J* = 8.0 Hz, 1H, Ar-H), 7.85 - 7.92 (m, 2H, Ar-H and H-6), 8.14 (d, *J* = 2.0 Hz, 1H, H-8), 8.53 (d, *J* = 7.0 Hz, 1H, H-2), 8.85 (d, *J* = 9.0 Hz, 1H, H-5), 11.20 (br s, 1H, NH). ^13^C-NMR (100 MHz, DMSO-*d*_6_) δ ppm 43.4 (CH_2_), 55.0 (OCH_3_), 63.8, 100.5, 114.3, 116.1 (C), 119.6, 121.8, 122.4 (C), 124.3, 124.4, 125.9, 127.3, 128.7, 129.2, 129.6, 130.0, 134.3 (C), 137.1 (C), 138.3 (C), 138.5 (C), 139.5 (C), 140.4 (C), 140.9 (C), 143.8, 144.7 (C), 154.7 (C). MS (70 eV) *m*/*z* (%): 538 (1, M^+^), 419 (100), 280 (22), 243 (18), 218 (54), 126 (24), 99 (24). Anal. Calcd. For C_31_H_24_Cl_2_N_4_O: C, 69.02; H, 4.48; N, 10.39. Found: C, 69.11; H, 4.55; N, 10.41.

*7-Chloro-N-(3-(1-(4-chlorophenyl)-5-(3,4,5-trimethoxyphenyl)-4,5-dihydro-1H-pyrazol-3-yl)phenyl)quinolin-4-amine* (**37**). Yellow solid; 53% yield; mp: 200–201 °C. FTIR (KBr) υ(cm^−1^): 3204 (NH), 3053 (=C-H), 1615 and 1589 (C=N and C=C). ^1^H-NMR (400 MHz, DMSO-*d*_6_) δ ppm 3.08–3.19 (m, 1H, H-4′a), 3.71 (s, 3H, OCH_3_), 3.79 (s, 6H, 2 × OCH_3_), 3.92 (dd, *J* = 17.7, 12.2 Hz, 1H, H-4′b), 5.51 (dd, *J* = 12.2, 6.0 Hz, 1H, H-5′), 6.82–6.93 (m, 1H, H-3), 6.99 (m, 2H, Ar-H), 7.17–7.20 (m, 4H, Ar-H), 7.51 (d, *J* = 7.8 Hz, 1H, Ar-H), 7.57–7.72 (m, 1H, Ar-H), 7.83–7.93 (m, 3H, Ar-H), 8.15 (d, *J* = 9.2 Hz, 1H, H-6), 8.54 (d, *J* = 6.8 Hz, 1H, H-2), 8.85 (d, *J* = 9.2 Hz, 1H, H-5), 11.19 (br s, 1H, NH). ^13^C-NMR (100 MHz, DMSO-*d*_6_) δ ppm 43.5 (CH_2_), 56.4 (2 × OCH_3_), 60.5 (OCH_3_), 64.3, 100.0 (C), 101.0, 103.4, 114.1, 114.5, 115.1, 116.4 (C), 119.7, 122.8, 123.2 (C), 126.4, 128.1, 129.0, 129.2, 134.4 (C), 136.4 (C), 137.1 (C), 137.2 (C), 138.1 (C), 139.4 (C), 143.9, 148.1 (C), 153.8 (C), 155.5 (C). MS (70 eV) *m*/*z* (%): 598 (100, M^+^), 431 (59), 279 (16), 125 (39), 90 (10). Anal. Calcd. For C_33_H_28_Cl_2_N_4_O_3_: C, 66.11; H, 4.71; N, 9.35. Found: C, 66.15; H, 4.78; N, 9.45. 

*7-Chloro-N-(3-(1-(4-chlorophenyl)-5-(p-tolyl)-4,5-dihydro-1H-pyrazol-3-yl)phenyl)quinolin-4-amine* (**38**). Yellow solid; 60% yield; mp: 207–208 °C. FTIR (KBr) υ(cm^−1^): 3174 (NH), 3050 (=C-H), 1618 and 1590 (C=N and C=C). ^1^H-NMR (400 MHz, DMSO-*d*_6_) δ ppm 2.25 (s, 3H, CH_3_), 3.13 (dd, *J* = 17.6, 6.0 Hz, 1H, H-4′a), 3.94 (dd, *J* = 17.6, 12.3 Hz, 1H, H-4′b), 5.52 (dd, *J* = 12.3, 6.0 Hz, 1H, H-5′), 6.86 (d, *J* = 6.9 Hz, 1H, H-3), 6.99 (d, *J* = 8.8 Hz, 2H, Ar-H), 7.11–7.21 (m, 6H, Ar-H), 7.50 (d, *J* = 7.9 Hz, 1H, Ar-H), 7.62 (t, *J* = 7.9 Hz, 1H, Ar-H), 7.73 (d, *J* = 7.9 Hz, 1H, Ar-H), 7.84–7.92 (m, 2H, Ar-H and H-6), 8.20 (s, 1H, H-8), 8.53 (d, *J* = 6.9 Hz, 1H, H-2), 8.91 (d, *J* = 9.0 Hz, 1H, H-5), 11.27 (br s, 1H, NH). ^13^C-NMR (100 MHz, DMSO-*d*_6_) δ ppm 20.7 (CH_3_), 43.0 (CH_2_), 63.0, 100.5, 114.5, 116.0 (C), 119.3, 122.3, 122.4 (C), 124.9, 125.5, 125.8, 126.2, 127.4, 128.7, 129.6, 130.4, 134.0 (C), 136.8 (C), 137.5 (C), 138.4 (C), 138.9 (C), 139.1 (C), 142.7 (C), 143.5, 147.2 (C), 154.9 (C). MS (70 eV) *m*/*z* (%): 522 (100, M^+^), 431 (24), 279 (16), 125 (30), 91 (12), 44 (14). Anal. Calcd. For C_31_H_24_Cl_2_N_4_: C, 71.13; H, 4.62; N, 10.70. Found: C, 71.18; H, 4.74; N, 10.80. 

### 3.3. Antiproliferative Activity

The human tumor cell lines of the cancer screening panel were grown in an RPMI-1640 medium containing 5% fetal bovine serum and 2 mM l-glutamine. For a typical screening experiment, cells were inoculated into 96-well microtiter plates. After cell inoculation, the microtiter plates were incubated at 37 °C, 5% CO_2_, 95% air, and 100% relative humidity for 24 h prior to the addition of the tested compounds. After 24 h, two plates of each cell line were fixed in situ with TCA, to represent a measurement of the cell population for each cell line at the time of sample addition (Tz). The samples were solubilized in dimethyl sulfoxide (DMSO) at 400-fold the desired final maximum test concentration and stored frozen prior to use. At the time of compound addition, an aliquot of frozen concentrate was thawed and diluted to twice the desired final maximum test concentration with complete medium containing 50 μg/mL gentamicin. An additional four 10-fold or ½ log serial dilutions were made to provide a total of five drug concentrations plus the control. Aliquots of 100 μL of these different sample dilutions were added to the appropriate microtiter wells already containing 100 μL of medium, resulting in the required final sample concentrations [19]. After the tested compounds were added, the plates were incubated for an additional 48 h at 37 °C, 5% CO_2_, 95% air, and 100% relative humidity. For adherent cells, the assay was terminated by the addition of cold TCA. Cells were fixed in situ by the gentle addition of 50 μL of cold 50% (*w*/*v*) TCA (final concentration, 10% TCA) and incubated for 60 min at 4 °C. The supernatant was discarded, and plates were washed five times with tap water and air dried. Sulforhodamine B (SRB) solution (100 μL) at 0.4% (*w*/*v*) in 1% acetic acid was added to each well, and plates were incubated for 10 min at room temperature. After staining, unbound dye was removed by washing five times with 1% acetic acid and the plates were air dried. Bound stain was subsequently solubilized with 10 mM trizma base, and the absorbance was read on an automated plate reader at a wavelength of 515 nm. Using the seven absorbance measurements [time zero (Tz), control growth in the absence of drug (C), and test growth in the presence of drug at the five concentration levels (Ti)], the percentage growth was calculated at each of the drug concentrations levels. Percentage growth inhibition was calculated as: [(Ti − TZ)/(C − TZ)] × 100 for concentrations for which Ti > Tz, and [(Ti − TZ)/TZ] × 100 for concentrations for which Ti < Tz. Two dose-response parameters were calculated for each compound. Growth inhibition of 50% (GI_50_) was calculated from [(Ti − TZ)/(C − TZ)] × 100 = 50, which is the drug concentration resulting in a 50% lower net protein increase in the treated cells (measured by SRB staining) as compared to the net protein increase seen in the control cells and the LC_50_ (concentration of drug resulting in a 50% reduction in the measured protein at the end of the drug treatment as compared to that at the beginning), indicating a net loss of cells; calculated from [(Ti − TZ)/TZ] × 100 = −50). Values were calculated for each of these two parameters if the level of activity is reached; however, if the effect was not reached or was exceeded, the value for that parameter was expressed as greater or less than the maximum or minimum concentration tested [19,20,21].

### 3.4. Antifungal Activity

#### 3.4.1. Microorganisms and Media

For the antifungal evaluation, strains from the American Type Culture Collection (ATCC, Rockville, MD, USA), *C. albicans* ATCC 10231 and *C. neoformans* ATCC 32264, were used. Strains were grown on Sabouraud-chloramphenicol agar slants for 48 h at 30 °C, maintained on slopes of Sabouraud-dextrose agar (SDA, Oxoid, Basingstoke Hampshire, UK), and sub-cultured every 15 days to prevent pleomorphic transformations. Inocula were obtained according to reported procedures [26] and adjusted to 1–5 × 10^3^ cells with colony-forming units (CFU)/mL.

#### 3.4.2. Fungal Growth Inhibition Percentage Determination

Yeast broth microdilution technique M27-A3 of CLSI [26] was performed in 96-well microplates. For the assay, compound test-wells (CTWs) were prepared with stock solutions of each compound in DMSO (maximum concentration ≤1%), diluted with RPMI-1640 to final concentrations of 250–3.9 μg/mL. An inoculum suspension (100 μL) was added to each well (final volume in the well = 200 μL). A growth control well (GCW) (containing medium, inoculum, and the same amount of DMSO used in a CTW, but compound-free) and a sterility control well (SCW) (sample, medium, and sterile water instead of inoculum) were included for each fungus tested. Microtiter trays were incubated in a moist, dark chamber at 30 °C for 48 h for both yeasts. Microplates were read in a VERSA Max microplate reader (Molecular Devices, Sunnyvale, CA, USA). Amphotericin B (Sigma-Aldrich, St. Louis, MO, USA) was used as a positive control. Tests were performed in triplicate. Reduction of growth for each compound concentration was calculated as follows: % of inhibition = 100 − (OD 405 CTW − OD 405 SCW)/(OD 405 GCW − OD 405 SCW). The means ± SD (standard deviations) were used for constructing the dose–response curves. Representing % inhibition vs. concentration of each compound. Dose–response curves were constructed with SigmaPlot 11.0 software (Systat Software Inc., San Jose, CA, USA).

#### 3.4.3. MIC_100_, MIC_80_, and MIC_50_ Determinations

Three endpoints were defined from the dose–response curves. Minimum Inhibitory concentration (MIC) resulting in total fungal growth inhibition was named MIC_100_ while MIC_80_ and MIC_50_ were defined as the minimum concentration that inhibits 80% or 50% of the fungal growth, respectively.

#### 3.4.4. Statistical Analysis

Comparisons of activities were statistically analyzed by applying the Student *t*-test. Values of *p* < 0.05 were considered to be significant.

## 4. Conclusions

In this article, we report the synthesis of a novel series of *N*-aryl-substituted pyrazolines under mild reaction conditions, easy work-up, short reaction times, and good yields. The antiproliferative evaluation data against 58 cancer cell lines revealed that compounds with an *m-*substitution in ring **A** and EDG (4-OCH_3_ and 3,4,5-*tri*-OCH_3_) in ring **C** exhibited the highest activity, with GI_50_ values lower than 1.0 µM for several of the cell lines tested (Compounds **25**, **30**, **31**, **36**, and **37**). Furthermore, these compounds displayed higher cytostatic activity against several cell lines compared to the standard drug, adriamycin. Regarding the antifungal activity, compounds **15**–**38** showed activity against *C. albicans* and *C. neoformans* with varied MIC_50_ values between <3.9 and 250 µg/mL. However, among the different sub-groups, the compounds in series (ii) showed better activity than those in group (i) against both fungi, These results indicate that the position of the azole moiety does play a role in the antifungal activity and that compounds with this moiety in the *m*-position are better antifungal candidates than those with the azole moiety in the *p*-position. Within the two sub-groups in series (ii), the best activity was displayed by compounds with a *p*-Cl moiety in the aryl ring **D**. It could be showed that *C. neoformans* had higher sensitivity to these compounds than the other fungus tested (*C. albicans)*. Compound **36** exhibited the highest antifungal properties, becoming an interesting candidate for new antifungal studies.

## Figures and Tables

**Table 1:** Synthesis of pyrazolines (**15**–**38**) via cyclocondensation reactions.

R, R′	Br, H	Cl, H	H, H	OCH_3_, H	OCH_3_, OCH_3_	CH_3_, H
**Compound Number and Isolated Yield (%)**	**15** (90)	**16** (92)	**17** (83)	**18** (77)	**19** (76)	**20** (86)
	**21** (80)	**22** (87)	**23** (73)	**24** (67)	**25** (63)	**26** (70)
	**27** (83)	**28** (81)	**29** (73)	**30** (68)	**31** (65)	**32** (72)
	**33** (75)	**34** (71)	**35** (61)	**36** (47)	**37** (53)	**38** (60)

**Table 2:** In vitro cytotoxic activities of compounds **25**, **30**, **31**, **36**, and **37**, expressed as growth inhibition and lethal concentration of cancer cell lines and compared with the standard drug adriamycin **^a^**.

Panel Cell Line	Compounds										Doxorubicin (adriamycin) NSC 123127	
	25		30		31		36		37		100 µM ^d^	
	GI_50_^b^ (µM)	LC_50_^c^ (µM)	GI_50_ (µM)	LC_50_ (µM)	GI_50_ (µM)	LC_50_ (µM)	GI_50_ (µM)	LC_50_ (µM)	GI_50_ (µM)	LC_50_ (µM)	GI_50_ (µM)	LC_50_ (µM)
***Leukemia***												
CCRF-CEM	2.50	>100	**0.55**	>100	6.40	>100	1.84	>100	4.84	>100	0.08	100.00
HL-60(TB)	4.68	>100	**0.28**	>100	5.37	>100	4.78	>100	4.18	>100	0.12	89.33
K-562	**0.86**	>100	**0.49**	>100	3.75	>100	2.75	>100	**0.76**	>100	0.19	100.00
MOLT-4	3.86	>100	**0.43**	>100	7.98	>100	3.18	>100	3.68	>100	0.03	100.00
RPMI-8226	----	----	1.27	99.1	5.33	>100	4.70	>100	3.69	>100	0.08	100.00
SR	1.20	>100	1.17	89.7	5.49	>100	4.29	>100	2.54	>100	0.03	100.00
***Non-small Cell Lung***												
A549/ATCC	4.15	>100	2.00	>100	8.87	>100	4.70	>100	2.72	>100	0.06	100.00
HOP-62	2.14	>100	**0.95**	>100	3.81	>100	2.44	>100	1.39	>100	0.07	67.61
HOP-92	1.64	>100	3.93	>100	3.60	>100	10.8	>100	1.85	>100	0.10	42.27
NCI-H226	4.77	>100	11.3	>100	41.2	>100	14.2	>100	8.46	>100	0.05	6.40
NCI-H23	8.82	>100	8.78	>100	64.4	>100	17.5	>100	17.5	>100	0.15	13.15
NCI-H322M	13.2	>100	6.39	>100	53.3	>100	15.2	>100	22.2	>100	0.54	67.76
NCI-H460	3.16	>100	**0.42**	47.0	3.27	>100	3.04	>100	**0.77**	>100	0.02	51.29
NCI-H522	1.85	>100	**0.95**	57.0	3.60	>100	4.09	80.8	1.57	>100	0.03	2.80
***Colon Cancer***												
COLO 205	3.28	>100	1.43	48.5	15.7	>100	3.78	>100	3.13	>100	0.18	4.33
HCC-2998	2.09	>100	**0.54**	47.3	5.32	>100	3.41	80.4	3.14	>100	0.26	21.68
HCT-116	3.08	>100	1.12	43.3	6.51	>100	2.27	56.4	1.68	>100	0.08	54.58
HCT-15	**0.88**	>100	**0.34**	57.6	2.91	>100	2.30	>100	1.28	>100	6.46	100.00
HT29	2.66	>100	----	----	----	----	----	----	----	----	0.12	67.45
KM12	1.67	>100	**0.23**	>100	2.00	>100	**0.71**	>100	**0.82**	>100	0.27	92.68
SW-620	2.55	>100	0.28	47.1	2.44	>100	**0.53**	93.6	**0.67**	>100	0.09	58.61
***CNS Cancer***												
SF-268	5.49	>100	4.19	>100	75.5	>100	15.5	>100	8.16	>100	0.10	30.48
SF-295	2.02	>100	1.62	60.4	3.55	>100	5.80	65.2	1.71	>100	0.10	69.98
SF-539	1.74	>100	1.15	9.23	3.08	>100	1.80	23.0	1.71	34.2	0.12	27.23
SNB-19	4.91	>100	6.90	>100	>100	>100	15.9	>100	30.3	>100	0.04	49.77
SNB-75	1.11	>100	3.34	>100	6.60	>100	12.5	>100	2.22	>100	0.07	3.30
U251	2.91	>100	----	----	----	----	----	----	----	----	0.04	30.62
***Melanoma***												
LOX IMVI	1.66	>100	0.34	34.8	2.30	>100	1.30	44.3	**0.65**	>100	0.07	50.35
MALME-3M	>100	>100	10.7	>100	>100	>100	22.7	>100	21.9	>100	0.12	3.97
M14	2.01	>100	**0.33**	>100	2.60	>100	**0.75**	47.8	**0.63**	>100	0.18	4.05
MDA-MB-435	**0.33**	>100	**0.22**	>100	1.47	>100	**0.72**	>100	**0.80**	>100	0.25	9.57
SK-MEL-2	2.35	>100	2.07	>100	8.78	>100	11.5	>100	4.65	>100	0.17	1.06
SK-MEL-28	2.66	>100	4.41	>100	10.4	>100	7.07	>100	4.54	>100	0.21	15.92
SK-MEL-5	2.68	>100	1.46	18.7	4.56	96.6	3.01	33.4	1.37	18.5	0.08	0.49
UACC-257	2.72	>100	1.36	>100	10.5	>100	6.40	>100	3.45	>100	0.14	8.15
UACC-62	1.17	>100	**0.05**	54.9	**0.87**	>100	**0.60**	54.0	**0.31**	>100	0.12	0.74
***Ovarian Cancer***												
IGROV1	4.81	>100	11.6	>100	29.2	>100	18.8	>100	3.89	>100	0.17	100.00
OVCAR-3	1.88	>100	3.20	>100	8.64	>100	11.0	>100	3.72	>100	0.39	84.33
OVCAR-5	6.08	>100	11.8	>100	>100	>100	15.6	>100	13.2	>100	0.41	100.00
OVCAR-8	4.19	>100	2.85	>100	7.68	>100	5.00	>100	5.36	>100	0.10	43.25
NCI/ADR-RES	1.70	>100	1.51	>100	5.97	>100	9.51	>100	3.18	>100	7.16	100.00
SK-OV-3	3.26	>100	9.62	>100	>100	>100	15.0	>100	17.3	>100	0.22	100.00
***Renal Cancer***												
786-0	2.88	>100	3.95	>100	5.88	>100	2.94	>100	2.05	>100	0.13	51.64
A498	1.66	>100	2.87	>100	-	>100	4.64	>100	4.65	>100	0.10	1.90
ACHN	1.85	>100	3.50	>100	3.52	>100	4.63	>100	1.48	>100	0.08	100.00
CAKI-1	2.12	>100	**0.68**	>100	2.52	>100	2.38	>100	**0.69**	>100	0.95	100.00
RXF 393	1.34	>100	8.91	>100	8.37	>100	17.5	>100	2.42	>100	0.10	4.69
SN12C	7.56	>100	7.19	>100	>100	>100	13.0	>100	7.24	>100	0.07	72.44
TK-10	5.05	>100	3.76	>100	9.57	>100	12.9	>100	3.27	>100	0.18	86.70
UO-31	1.58	>100	**0.48**	>100	1.68	>100	1.87	>100	**0.49**	>100	0.49	26.18
***Prostate Cancer***												
PC-3	1.47	>100	**0.45**	>100	1.71	>100	1.18	>100	**0.82**	>100	0.32	87.10
DU-145	8.08	>100	14.0	>100	>100	>100	28.4	>100	17.1	>100	0.11	100.00
***Breast Cancer***												
MCF7	**0.86**	>100	1.52	>100	2.74	>100	3.45	>100	2.31	>100	0.03	51.29
MDA-MB-231/ATCC	1.93	>100	1.31	>100	4.77	>100	4.84	>100	2.97	>100	0.51	34.75
HS 578T	2.18	>100	6.79	>100	9.22	>100	14.7	>100	2.45	>100	0.33	85.70
BT-549	2.93	>100	7.42	>100	13.9	>100	2.67	>100	4.63	>100	0.23	21.33
T-47D	2.13	>100	**0.17**	>100	1.44	>100	**0.70**	>100	**0.69**	>100	0.06	85.70
MDA-MB-468	**0.91**	>100	2.13	>100	20.1	>100	4.45	>100	6.29	>100	0.05	2.52

^a^ Data obtained from NCI’s in vitro disease-oriented human tumor cell lines screen; ^b^ GI_50_ was the drug concentration resulting in a 50% reduction in the net protein increase (as measured by SRB staining) in control cells during the drug incubation. Determined at five concentration levels (100, 10, 1.0, 0.1, and 0.01 µM); ^c^ LC_50_ a parameter of cytotoxicity and reflects the molar concentration needed to kill 50% of the cells; ^d^ The values of activity against human tumor cell lines displayed by mycin correspond to the reported by NCI at highest concentration of $10^{-4}$ M. Please visit: http://dtp.nci.nih.gov/dtpstandard/cancerscreeningdata/index.jsp. The most active compounds were highlighted in grey.

**Table 3:**
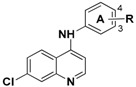
Antifungal activity (MIC_100_, MIC_80_, MIC_50_ values) obtained for compounds **15**–**38** against the fungi *Candida albicans* and *Cryptococcus neoformans*.

Type	Sub-Type	Compound	Position on Ring A	R	Fungus	MIC_100_	MIC_80_	MIC_50_
**i**	**i.1**	**15**	4	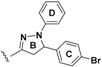	*Ca*	>250	250	250
					*Cn*	>250	250	125
		**16**	4	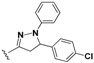	*Ca*	>250	250	250
					*Cn*	>250	250	125
		**17**	4	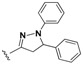	*Ca*	>250	250	250
					*Cn*	>250	250	125
		**18**	4	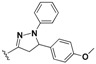	*Ca*	>250	250	250
					*Cn*	>250	250	250
		**19**	4	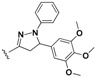	*Ca*	>250	250	62.5
					*Cn*	>250	>250	250
		**20**	4	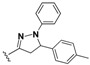	*Ca*	>250	125	31.2
					*Cn*	>250	250	125
	**i.2**	**21**	4	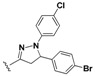	*Ca*	>250	250	62.5
					*Cn*	>250	250	125
		**22**	4	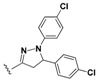	*Ca*	>250	>250	250
					*Cn*	>250	250	62.5
		**23**	4	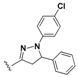	*Ca*	>250	62.5	31.2
					*Cn*	>250	250	250
		**24**	4	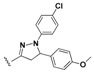	*Ca*	250	250	31.2
					*Cn*	>250	>250	>250
		**25**	4	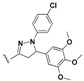	*Ca*	250	125	15.6
					*Cn*	62.5	31.2	15.6
		**26**	4	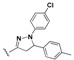	*Ca*	>250	>250	250
					*Cn*	250	125	125
**ii**	**ii.1**	**27**	3	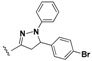	*Ca*	250	125	62.5
					*Cn*	>250	>250	125
		**28**	3	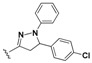	*Ca*	>250	250	62.5
					*Cn*	>250	>250	62.5
		**29**	3	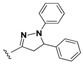	*Ca*	>250	>250	125
					*Cn*	>250	>250	250
		**30**	3	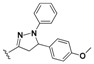	*Ca*	250	250	125
					*Cn*	250	250	31.2
		**31**	3	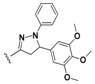	*Ca*	>250	>250	250
					*Cn*	>250	>250	250
		**32**	3	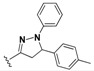	*Ca*	62.5	62.5	15.6
					*Cn*	31.2	15.6	7.8
	**ii.2**	**33**	3	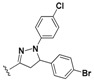	*Ca*	>250	>250	250
					*Cn*	250	250	62.5
		**34**	3	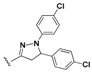	*Ca*	>250	>250	>250
					*Cn*	>250	250	31.2
		**35**	3	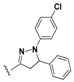	*Ca*	>250	250	125
					*Cn*	>250	250	62.5
		**36**	3	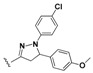	*Ca*	62.5	62.5	15.6
					*Cn*	<3.9	<3.9	<3.9
		**37**	3	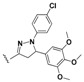	*Ca*	125	31.2	31.2
					*Cn*	125	62.5	31.2
		**38**	3	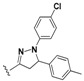	*Ca*	125	125	62.5
					*Cn*	125	62.5	62.5
		Amphotericin B			*Ca*	1.00	0.50	0.50
					*Cn*	1.25	0.50	0.50
